# Aging augments obesity-induced thymic involution and peripheral T cell exhaustion altering the “obesity paradox”

**DOI:** 10.3389/fimmu.2022.1012016

**Published:** 2023-01-26

**Authors:** Logan V. Vick, Craig P. Collins, Lam T. Khuat, Ziming Wang, Cordelia Dunai, Ethan G. Aguilar, Kevin Stoffel, Sai Yendamuri, Randall Smith, Sarbajit Mukherjee, Joseph Barbi, Robert J. Canter, Arta M. Monjazeb, William J. Murphy

**Affiliations:** ^1^ Department of Dermatology, University of California Davis School of Medicine, Sacramento, CA, United States; ^2^ Department of Thoracic Surgery, Roswell Park Comprehensive Cancer Center, Buffalo, NY, United States; ^3^ Department of Immunology Roswell Park Comprehensive Cancer Center, Buffalo, NY, United States; ^4^ Department of Medicine, Roswell Park Comprehensive Cancer Center, Buffalo, NY, United States; ^5^ Division of Surgical Oncology, Department of Surgery, University of California Davis Comprehensive Cancer Center, University of California Davis School of Medicine, Sacramento, CA, United States; ^6^ Department of Radiation Oncology, University of California Davis Comprehensive Cancer Center, University of California School of Medicine, Sacramento, CA, United States; ^7^ Department of Internal Medicine, Division of Hematology and Oncology, University of California Davis School of Medicine, Sacramento, CA, United States

**Keywords:** obesity, T cell, immunotherapy, inflammaging, aging, thymic involution, PD-1

## Abstract

**Introduction:**

The incidence of obesity, a condition characterized by systemic chronic inflammation, has reached pandemic proportions and is a poor prognostic factor in many pathologic states. However, its role on immune parameters has been diverse and at times contradictory. We have previously demonstrated that obesity can result in what has been called the “obesity paradox” which results in increased T cell exhaustion, but also greater efficacy of immune checkpoint blockade in cancer treatment.

**Methods:**

The role of obesity, particularly in the context of aging, has not been robustly explored using preclinical models. We therefore evaluated how age impacts the immune environment on T cell development and function using diet-induced obese (DIO) mice.

**Results:**

We observed that DIO mice initially displayed greater thymopoiesis but then developed greater thymic involution over time compared to their lean counterparts. Both aging and obesity resulted in increased T cell memory conversion combined with increased expression of T cell exhaustion markers and Treg expansion. This increased T cell immunosuppression with age then resulted in a loss of anti-tumor efficacy by immune checkpoint inhibitors (ICIs) in older DIO mice compared to the younger DIO counterparts.

**Discussion:**

These results suggest that both aging and obesity contribute to T cell dysfunction resulting in increased thymic involution. This combined with increased T cell exhaustion and immunosuppressive parameters affects immunotherapy efficacy reducing the advantage of obesity in cancer immunotherapy responses.

## Introduction

Obesity is defined by the accumulation of excessive body fat and is associated with poorer health outcomes overall. These include decreased life expectancy and worse prognostic indicators for a wide spectrum of diseases including diabetes mellitus, cardiovascular disorders, osteoarthritis, and cancer (1–3). The prevalence of obesity has been on the rise with more than 1.9 billion adults worldwide being characterized as overweight and obese in 2015 (4). With such a large fraction of the population being afflicted by obesity the need to understand how this affliction influences disease progression, treatment, and prevention has become more pressing and relevant. Importantly, obesity’s connection with chronic low-grade inflammation has been described as a potential mediator of disease pathology by acting as a nonspecific stimulus for the immune system (5–8). Although, increased inflammation can lead to pathology it is not inherently detrimental and may also allow for proper immune functioning. This contributes to the “obesity paradox” in which some of the same processes affected by obesity can result in different outcomes contingent on the context, extent, and overall net effect. Obesity-associated inflammation also has been associated with increased aging parameters, referred to as “inflammaging” (9–11). This is believed to occur by a variety of mechanisms and affects every tissue and cell-type differently. With regard to effects on immune function, obesity has been generally regarded as promoting immunosuppressive pathways (12, 13) and displaying heightened pro-inflammatory responses to various pathogens or stimuli (14–16).

The impact of obesity on T cells has been under increased scrutiny due to the differential effects of obesity on not only T cell development but also hematopoiesis in general where both stimulatory and inhibitory effects have been reported (17–20). This duality of obesity centers around the presence of a nutrient-rich environment, including increases in IGF-1 but also the chronic pro-inflammatory state all of which affect immune status. All immune cell components, both with their development and function, are affected by obesity. With T cells, there has been considerable interest on effects upon the thymus. The thymus is critical for both T cell development and for naïve T cell output, but also undergoes involution with aging. However, using preclinical models, the effects of obesity on thymus size has been inconclusive with separate reports demonstrating seemingly contrary observations; one described increased thymus size in obesity while another reported increased thymic involution (21, 22). Notably, T cell function and obesity, has been more consistent with suppression. Both in humans and in diet indued obese (DIO) mouse models obesity has been associated with impaired T cell memory responses to different pathogens leading to poorer outcomes (23–25). Further, we have reported that obesity can both increase T cell memory conversion and generate T cells that exhibit an exhaustive phenotype (26). A significant factor in preclinical modeling of obesity centers on important variables such as the diet used to induce obesity and the time needed for this to occur.

Aging has also been demonstrated to markedly affect T cell development and function. In particular, aging is associated relatively early on with a reduction of naïve T cell numbers due to thymic involution (27–30). This then leads to an increased reliance on long-lived memory T cells for responses, yet aging is also associated with increased T cell dysfunction due to expression of exhaustion markers (i.e., PD-1, TIM3, LAG3) which dampen effector functions. The question arises as to the effects of obesity over time on T cell responses, particularly when evaluating cancer immunotherapy.

Thus, the understanding of how obesity directly contributes to aging and how these two mediators of inflammation modulate the immunes system in tandem still requires elucidation. We have previously reported that obesity can be seen as a paradox in immunotherapy treatment; where obesity promotes tumorigenesis and yet also results in greater cancer immunotherapy efficacy by checkpoint blockade in bothhumans and mouse preclinical models (26). In this study we build on our understandings of obesity by widening our scope considering questions of how aging and obesity are intertwined. To answer these questions, we used the DIO model looking at various time points following exposure to HFD. We report that DIO mice initially had greater thymic cellularity but underwent a more rapid and significantly greater thymic involution over time. We also observed increased memory T cell and Treg content in the DIO mice which also displayed a greater exhaustive phenotype. Notably, this observed exhaustive phenotype in DIO mice significantly increased with aging compared to lean counterparts. More importantly, we observed that this increased exhaustive phenotype in older DIO mice resulted in loss of anti-tumor efficacy using immune checkpoint blockade compared to younger DIO mice demonstrating potential limits regarding the advantages of obesity in the “obesity paradox”. These studies demonstrate that the impact of obesity on T cell status and function is also contingent on age.

## Methods

### Mice

Male C57BL/6 mice were obtained from Jackson or Taconic farms and housed under specific-pathogen-free conditions in animal facilities at The University of California Davis or at Roswell Park. All animal protocols were approved by the UC Davis or Roswell Park Institutional Animal Care and Use Committee (IACUC) and all studies adhered to the ethical standards set by the respective IACUC. Control and DIO mice were generated through use of open-source feeding of either 10% fat (D12450J and S4031) or 60% fat (D12492 and S3282) diets (obtained from Research Diets Inc/Bio-Serv). Once mice reached the ages of 6- to 8- weeks old special diets were initiated and mice remained on their respective diets continuously following diet initiation. Mice were weighed regularly, and weights were collected for each cohort as a part of routine monitoring.

### Tumor cell lines and treatment

Lewis Lung Carcinoma (3LL, CRL-1642) was obtained from the American Type Culture Collection (ATCC). Control and DIO C57BL/6 mice were challenged *via* subcutaneous injection in right hind flank with 1x10^6^ 3LL cells suspended in 100ul of PBS. Mice challenged with tumor received daily monitoring and tumors were measured using calipers every 2-3 days. Tumor volume was calculated using the equation length (mm) x width^2^ (mm^2^) x 0.5. In experiments where tumor bearing mice received treatment mice were administered a loading dose of 500ug of either anti-PD-1 (Clone: 29 F.1A12, BioXCell) or rat IgG (Jackson ImmunoResearch Laboratories, Inc.) intraperitoneal in 200ul of PBS. Subsequent doses were given every other day following initial loading dose for a total of six doses.

### Antibodies and flow cytometry (mouse)

Staining was performed on single cell suspensions of spleen, thymus, tumor, and mouse peripheral blood. Tissues were harvested and mechanically homogenized and filtered in preparation for staining. Cells were stained for viability using Zombie Red (BioLegend), Zombie NIR (BioLegend) or Fixable Viability Dye 780 (Invitrogen). Fc blocking was performed by incubating cells with Fc Block (anti-CD16/32 clone 93) for 15 minutes followed by a surface stain of fluorochrome-conjugated monoclonal antibodies (a list is provided below) in staining buffer (phosphate-buffered saline + 3% Nu serum). For stains involving intracellular markers cells were permeabilized using Cytofix/Cytoperm (BD Biosciences) for 20 min and washed prior to use of intracellular antibodies or was performed with a FOXP3/transcription factor fixation/permeabilization kit (eBiosciences) according to the manufacturer’s specification. Antibodies: PB-anti-CD45 (30-F11), PB-anti-CD44 (IM7), APC-Cy7-anti-CD45 (30-F11), BV711-anti-CD4 (RM4–5), BV785-anti-CD3 (17A2), BV605-anti-CD8a (53–6.7), Alexa Fluor 700-anti-Ki67 (16A8), FITC-anti-PD-1 (RMP1–30), PE-anti-Tim3 (RMT3–23), PerCP-eFluor 710-anti-Lag3 (eBioC9B7W), and PE-Cy7-anti-CD62L (MEL-14). Additional antibody clones used include: CD45 (clone I3/2.3), CD4 (L3T4), CD8 (53-6.7), CD25 (PC61), PD-1 (29F.1A12) FoxP3(150D/E4) and Ki67 (11F6).

### Metabolomics

Blood was collected *via* tail vein bleed or cardiac puncture in accordance with UC Davis IACUC guidelines and serum was obtained through use of BD microtainer separation tubes (365967). Serum Glucose (Fisher Diagnostics Middletown, VA) and blood Hemogobin A1c (Diazyme Poway, CA) enzymatic assays were performed by UC Davis Mouse Metabolic Phenotyping Center (MMPC). Serum Leptin was measured using an electro-chemiluminescent assay (Meso Scale Discovery).

### Magnetic resonance imaging

All mice used for imaging were anesthetized using a combination of isoflurane and oxygen prior to scanning on a Biospec 70/30 7.0-Tesla small-animal magnetic resonance imaging (MRI) system (Bruker Biospin Inc.) using a 60-mm quadrature transmitter/receiver coil for whole-body imaging. Scanning protocols and procedures were done as previously described (31).

### Statistical analysis

Graphs and statistical analysis were prepared using Prism software (GraphPad Software Inc., CA USA). Data were expressed as mean ± s.e.m. For analysis of three or more groups, analysis of variance (ANOVA) was performed with a Bonferroni or Tukey *post-hoc* test, when appropriate. Analysis of differences between two normally distributed test groups was performed using the Student’s *t*-test. Correlations drawn between thymus cellularity and body weight were evaluated through use of a runs test. *P* values were considered statistically significant if *P* < 0.05. Statistical outliers were identified using Grubb’s test. Statistical differences in survival were determined by log rank (Mantel–Cox) analysis.

## Results

### Effects of HFD on weight gain and metabolism over time

**Figure 1:**
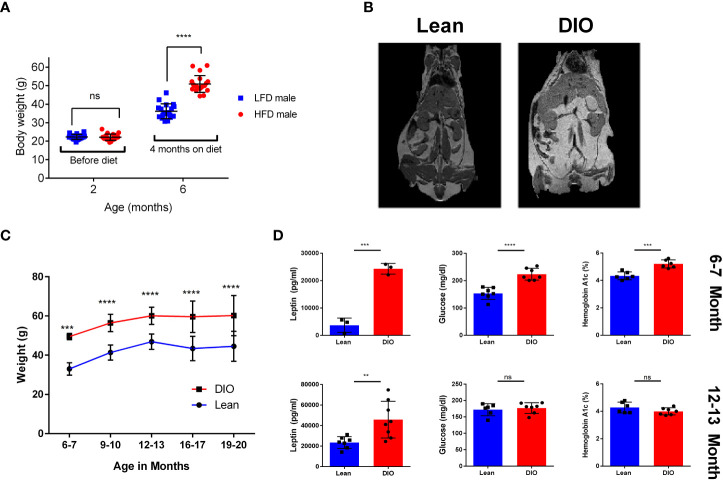
Effects of HFD on weight gain and metabolism over time. **(A)** Body Weights of two- and six-month C57BL/6 mice before and after Control Low Fat Diet (LFD) or High Fat Diet(HFD) (n =16-20/group). **(B)** Magnetic Resonance Imaging (MRI) of six-month Lean control and Diet induced obese (DIO) C57BL/6 mice. **(C)** Bodyweights of lean control or DIO mice overtime from ages of six to twenty months in age (n = 12-24/group). **(D)** Resting serum leptin, glucose, and blood hemoglobin A1c of six-seven month and twelve-thirteen-month lean control or DIO male C57BL/6 Mice (n = 3-8/group). Graphs depict error bars based on standard deviation. Data are representative of at least two independent experiments. Two-way ANOVA was used for analysis of **(A, C)**, unpaired student T test was used for assessments in **(D)**. **p < 0.01, ***p < 0.001, ****p < 0.0001, ns, not significant.

When assessing clinically obesity is often diagnosed through assessment of Body Mass Index (BMI); a positive diagnosis is considered any BMI greater than 30Kg/m^2^. When using mouse models to study obesity BMI is less feasible and criteria for obesity are often set either by weight, a weight differential compared to mice on a control diet or a combination of these two metrics. This can pose difficulties for modeling obesity and can make a study dependent upon the strain of mouse or model used as both the type of diet used and the diet duration can have significant effects. Examples of this can be seen with mouse strains such as BLAB/c, which we and others have reported to be resistant or have variable weight gain when placed on HFD (31, 32). Here we performed our assessment using male C57BL/6 mice placed on control or High Fat Diet (HFD) consisting of 60% fat by lard for a period of four months. We observed that, mice given HFD gained significantly more weight than control counterparts (**Figure 1A**). Further, to better determine the fat accumulation in DIO mice, we performed magnetic resonance imaging (MRI) which demonstrated significantly greater body fat content in DIO mice than control diet mice (**Figure 1B**). Many studies using DIO models are usually performed after several weeks to months on diet and used at approximately 6-7 months of age which is at variance with most preclinical studies using much younger mice. We therefore evaluated cohorts of C57BL/6 mice, maintained on control or HFD diets, from six to twenty months in age and observed that DIO mice maintained increased body weight even at advanced age despite notable increases in control mice (**Figure 1C**). Metabolic consequences of obesity include glucose intolerance and diabetes. Accordingly, glucose and Hemoglobin A1c were assessed and resulted in significantly increased glucose and Hemoglobin A1c in the six-seven-month DIO cohort (**Figure 1D**). Leptin levels were also observed to be higher in the DIO recipients regardless of age (**Figure 1D**). These observations confirm phenotypic differences between lean and DIO mice and that this differential in weight and adiposity is maintained with advanced age.

### Obesity induces early thymopoiesis but also greater thymic decline

**Figure 2:**
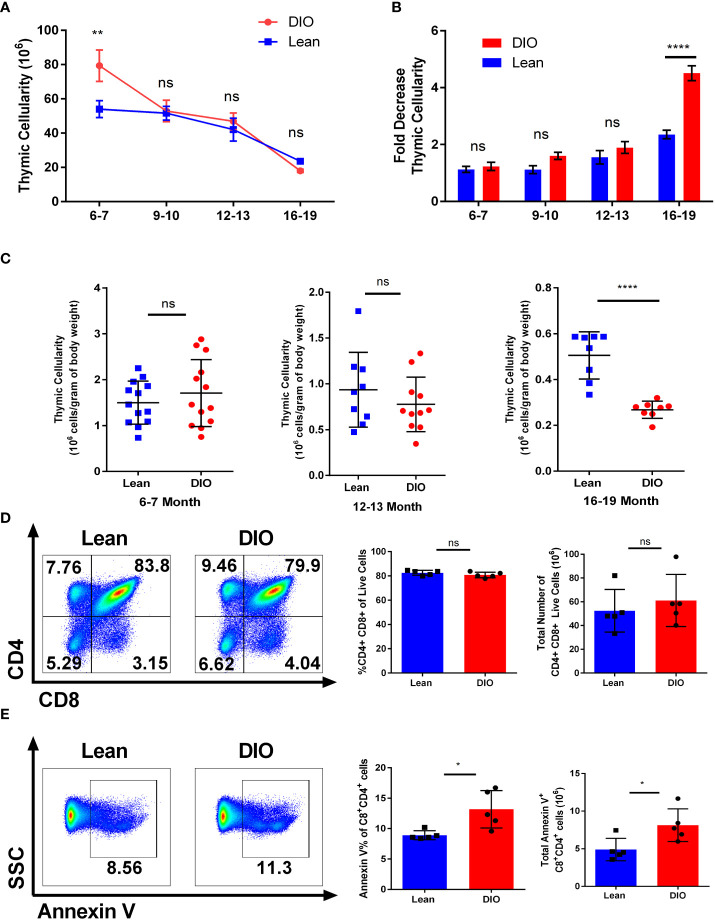
Obesity induces early thymopoiesis but also greater thymic decline. **(A)** Thymic cellularity in lean control or diet induced obese (DIO) C57BL/6 mice from ages of six to twenty months (n =8-16/group). **(B)** Fold change in Thymic involution of control and DIO mice normalized to thymic cellularity at six-seven months old (n =8-16/group). **(C)** Thymic cellularity of six-seven, twelve-thirteen-month, or sixteen-nineteen-month male C57BL/6 mice normalized to cellularity per gram of body weight. **(D)** Representative flow cytometry plots of CD4^+^ and CD8^+^ cells, percentages, and total numbers of the thymus in lean control and DIO six-seven-month C57BL/6 mice. **(E)** Representative flow cytometry plots, percentages, and total numbers of Annexin V staining on CD4/CD8 double positive cells in the thymus of C57BL/6 mice (n = 5/group). Data are representative of at least two independent experiments. Graphs in **(A)** depict error bars based on s.e.m and standard deviation for **(B–E)**. Two-way ANOVA was used for analysis of **(A, B)**, unpaired two tailed student T test was used for assessments in **(C-E)**. *p < 0.05, **p < 0.01, ****p < 0.0001, ns, not significant.

Conflicting reports using DIO mouse models identified contrasting data on the role obesity plays in both lymphopoiesis and thymopoiesis (21, 22). However, key differences between these reports involved both the diets and the age of the mice assessed which may have contributed to these seemingly contradictory outcomes. We performed an assessment of the relationship between thymic cellularity on DIO mice at different ages. Starting with mice as young as six-seven months we were able to confirm that at least early on, DIO mice presented with a larger thymus than their lean counterparts as determined by total thymus cellularity following mechanical processing (**Figure 2A**). However, this effect dissipated markedly over time. Significantly greater thymic involution was observed in the DIO mice by nineteen months with the DIO mice consistently demonstrating increased reduction. More than a sixfold decrease in cellularity by nineteen months was observed in DIO mice compared to the decreased involution and cell loss associated with lean control mice (**Figure 2B**). To validate our observation, we next evaluated the proportional impact of body weight on thymic cellularity. We did so by narrowing the assessment on six-seven month, twelve–thirteen-month, and sixteen-nineteen-month-old mice (**Supplemental Tables 1, 2**). From this we interrogated the correlation between body weight and thymic cellularity to help elucidate whether the larger size of the animal alone correlated with increased thymic cellularity (**Supplemental 1A**). We observed that in the younger six-seven-month groups DIO mice had greater weight and cellularity but did not have a greater proportional increase. Similarly, lean mice also did not demonstrate a significant association of increased thymic cellularity with increased body weight. Even in advanced age, at sixteen-nineteen-months the association of increased body weight with increased thymic cellularity was absent with body weight having no correlation on the size of the thymus. To better capture the relationship between obesity and thymic involution, we examined the proportional relationship of thymic cellularity per gram of total body weight (**Figure 2C**). From this we observed minimal differences in the thymic ratio to body weight at six-seven months, but we observed significant involution at advanced age where DIO mice underwent greater involution and had far fewer cells per total body weight. We then determined if the composition of the thymus differed between lean and DIO mice and whether this may have contributed to our observations in involution. We assessed through flow cytometry to delineate thymic T cells from thymic epithelium and were able to evaluate thymic sub populations. We observed minimal difference between lean and DIO CD4/CD8 double positive cells or other subset populations both by percentage and total numbers (**Figure 2D**; **Supplemental 1B, C**).

We then assessed the level of apoptosis of the thymocytes in DIO and control mice. Prior reports examining the thymus using obese mice deficient in leptin have been problematic given that leptin itself has been demonstrated to affect thymocyte survival, especially in stress situations (22, 33, 34). We observed significant differences in thymocyte apoptosis of six month lean and DIO mice with the latter demonstrating greater early apoptosis as determined by annexin V positivity (**Figure 2E**). Signs of apoptosis were observed most notably in the CD4/CD8 double positive subset (**Figures 2E**; **Supplemental 1D, E**). These data suggest obesity may have dual roles on T cell development in which at earlier stages the increased leptin and nutrient-rich environment promotes increased thymopoiesis. However, as the DIO mice age significantly greater thymic involution occurs which results in greater reduction of naïve T cell output.

### DIO mice demonstrate increased T cell memory cells which is further increased with aging

**Figure 3:**
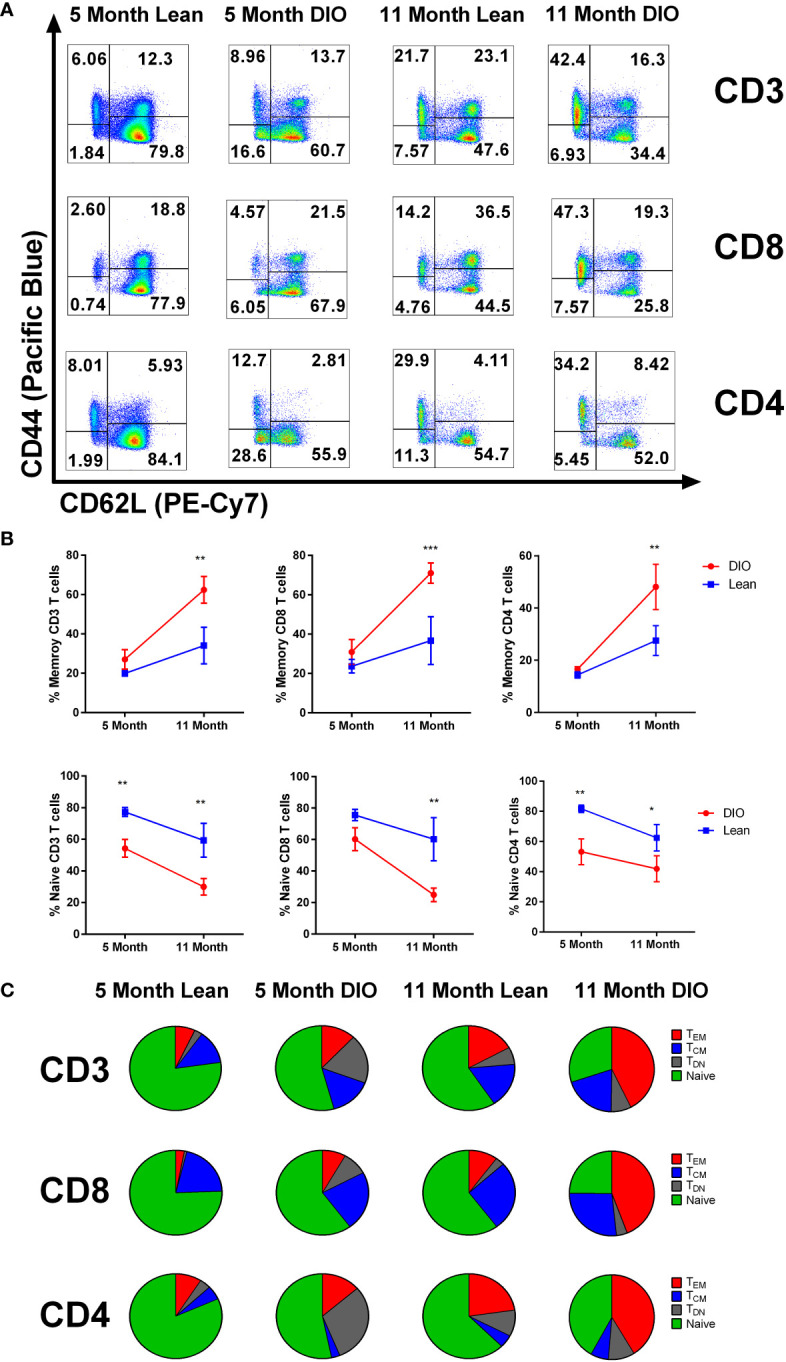
DIO mice demonstrate increased T cell memory cells which is further increased with aging. **(A)** representative flow cytometry of CD3, CD4 and CD8 positive T cells staining for CD44 and CD62L, and **(B)** CD44+/- frequencies from peripheral blood of lean control or Diet induce obese (DIO) five and eleven-month-old C57BL/6 mice (n = 3-4/group). **(C)** Central memory, effector memory and naïve CD3, CD4 and CD8 T cell population from peripheral blood of lean control or DIO five and eleven-month-old C57BL/6 mice depicted using pie charts. Data are representative of at least two independent experiments. Error bars ae based of standard deviation **(B)**. Two-way ANOVA was used for analysis of **(B)**. *p < 0.05, **p < 0.01, ***p < 0.001, ns, not significant.

We have previously reported that younger DIO mice have a greater proportion of CD44^+^ memory T cells than lean controls (26). In this study, we next determined whether this elevated memory conversion and phenotype in DIO mice increased or remained consistent over time. To examine this question, we collected the peripheral blood of five- and eleven-month DIO and control diet mice for immune phenotyping. T cell assessment *via* flow cytometry evaluating the central memory (CD44^+^ CD62L^+^), effector memory (CD44^+^ CD62L^-^), naïve (CD44^-^ CD62L^+^) and double negative (CD44^-^ CD62L^-^) compartments was performed (**Figure 3A**). The data confirmed elevated memory content in total CD3^+^ T cells particularly in the CD8 population of DIO mice. Not surprisingly in the older groups both lean and DIO mice increased in their overall memory T cell compartment, yet the extent of this increase was markedly different with large increases observed in older DIO mice while the naïve compartment significantly decreased (**Figures 3B, C**). Percentages and fold change in T cell subsets were all far higher in aged DIO mice than in lean counterparts with increases of nearly four times more memory conversion (**Supplemental Figures 2A, B**). These data confirm that DIO mice, both young and old, demonstrate increased T cell memory conversion. Further, while naïve to memory conversion occurs routinely during aging, this is accelerated under obesity.

### Aging and obesity both contribute to increased exhausted T-cell phenotypes and immunosuppressive Treg populations

**Figure 4:**
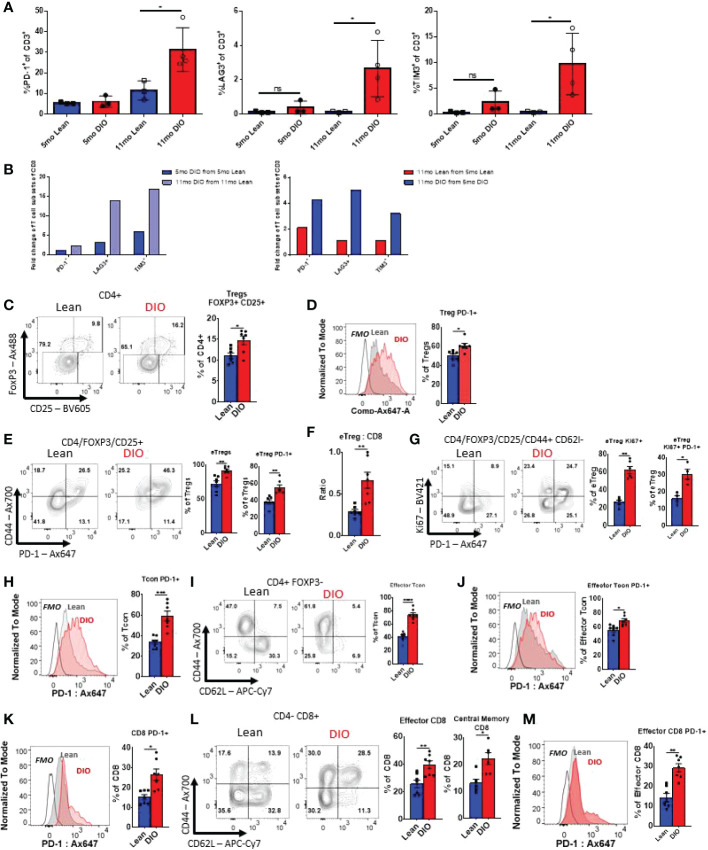
Aging and obesity both contribute to increased exhausted T-cell phenotypes and immunosuppressive Treg populations. **(A, B)** Flow cytometry frequencies of PD-1^+^, TIM3^+^ and Lag3 ^+^ CD3, CD4 and CD8 T cells from peripheral blood of Control or DIO five and eleven-month-old C57BL/6 mice (n = 3-4/group). **(B)** Fold change in CD3, CD4 and CD8 T cell populations of PD-1, Lag3, and Tim3 between Control/DIO Mice and from five- to eleven-month-old mice **(C–E)** Single-cell suspensions of splenocytes were generated for flow cytometry analysis, and the proportions of Foxp3+/CD25+ Regulatory T cells (“Tregs”) among the recovered viable CD4+ leukocytes (CD4+/CD45+ cells) were observed as were levels of PD-1 on these cells and the ratio of activated “effector” Tregs (or eTregs; CD4+/CD45+/Foxp3+/CD25+/CD44^high^/CD62L^low^) to CD8 T cells. **(F, G)** The frequencies of Tregs expressing activation-associated markers CD44, PD-1, and KI67 were observed as well as the fraction of the splenic Tregs displaying an eTreg profile. **(H–J)** The expression of PD-1 by conventional, non-regulatory CD4+ T cells (“Tcon” CD4+/CD45+/CD25-/Foxp3-) as well as the fraction of these cells displaying activated, effector surface marker profiles (CD44^high^/CD62L^low^) and the expression of PD-1 by effector Tcons was also determined by flow cytometry. **(K–M)** Similarly, the effects of obesity on PD-1 levels in the bulk CD8+ T cell (CD8+/CD4-/CD45+) and effector CD8+ pool were interrogated in addition to the relative frequencies of effector and memory (CD44^high^/CD62L^high^) CD8+ subsets. Shown are representative flow plots and quantified results from at least two independent experiments (n = 4-8/group). Error bars depict the SEM. *p<0.05, **p<0.01, ***p < 0.001, ****p < 0.0001 by unpaired t test.

We and others have previously demonstrated that obesity contributes to an increased exhaustive T cell phenotype (26, 35). T cell exhaustion a condition that arises in the presences of chronic antigen stimulation most notably during chronic viral infection or cancer is often defined by decreased proliferative capacity and functionality, but also through surrogate cellular surface markers of exhaustion including PD-1, Tim3, and Lag3 which have been demonstrated to be increased with aging (36–38). Reports have also identified obesity as a clear inducer of exhaustion marker expression, particularly in adipose tissue T cells (39). To evaluate the impact of obesity and advanced age in the periphery we again started through collection of peripheral blood. We assessed key T cell exhaustive markers including PD-1, Tim3 and Lag3 and evaluated both young and aged lean and DIO mice (**Figures 4A, B**). We observed that in peripheral blood five-month-old lean and DIO mice demonstrated minimal difference in exhaustive marker expression in CD3 T cells. Assessment of mice on control diets with increased age, demonstrated minor differences in exhaustive markers including increases in PD-1 expression. However, eleven-month DIO mice saw significant increases in all the exhaustive markers with levels that were three to four-fold higher than T cells from DIO mice at five months of age or with age-matched control mice. We further gated solely on memory CD3 T cells to determine if these increases in exhaustive markers were dependent on naïve T cell activation, and we observed that increased trends in exhaustive markers were maintained (**Supplemental Figure 3A**). These data indicate that aging and obesity in combination markedly augments an exhaustive phenotype compared to lean controls with increases in PD-1, Tim3, and Lag3.

We then assessed other T cell subsets associated with obesity and aging; notably CD4+ Tregs (40, 41). Flow cytometry analysis was performed on the spleens of five-month-old lean and DIO mice to determine the population of Tregs (CD45^+^CD3^+^CD4^+^FoxP3^+^CD25^+^) (**Supplemental Figure 3B**). Significantly elevated populations of Treg cells in DIO mice when comparing to lean mice (**Figure 4C**) were observed. Further analysis revealed that DIO mice had a higher proportion of Tregs displaying indicators of an activated effector phenotype. These included heightened expression of PD-1 in the general Tregs pool (**Figure 4D**), as well as Tregs displaying a CD44^high^ or CD44^high^CD62L^low^ profile (so called effector or “eTregs”), which were found more prominently in DIO mice (**Figure 4E**). An elevated ratio of eTregs to CD8 T cells (**Figures 4F**), indicative of heightened immunosuppressive function, was also found. These eTregs also displayed significantly heightened proliferation, as determined *via* Ki-67 staining, and both proliferating and non-proliferating Tregs expressed elevated PD-1 levels in DIO mice (**Figures 4F, G**). These data suggests that the obese environment supports Treg expansion and activation, potentially contributing to further immune suppression and T cell function impairment.

Next, we sought to determine how the obese environment impacted both CD4+ and CD8+ subsets in terms of expression of exhaustion markers. Five-month-old lean and DIO mice splenic CD4+ T conventional (Tcon) cells (CD45^+^CD3^+^CD4^+^CD8^-^CD25^-^Foxp3^-^) were analyzed *via* flow cytometry. The DIO mice were determined to have significantly higher levels of baseline PD-1 expression by Tcon cells (**Figure 4H**). It was also noted that in DIO mice a larger fraction of Tcons displayed an effector phenotype (CD44+/CD62L-; **Figures 4I**). These cells also showed heightened PD-1 positivity compared to those of lean controls (**Figure 4J**). The same phenomenon was observed in cytotoxic CD8 T cells (CD45^+^CD3^+^CD4^-^ CD8^+^), with both general CD8^+^ (**Figure 4K**), effector, and central memory T cells (**Figures 4L, M**) subsets displayed significantly elevated levels of PD-1 (**Figure 4K–M**). As in our analysis of circulating T cells, reduced naïve cell pools in both CD4+ cand CD8+ T cell compartments were seen among the splenocytes of DIO mice (**Figure 4I–L**). This data is indicative of obesity driving exhaustion in helper and cytotoxic T cell subsets, which could impair immune responses during an immune challenge suggesting potentially greater suppression with obesity which can be compounded by increased exhaustion with age.

### Aging ameliorates the increased anti-tumor efficacy of ICI in DIO mice

**Figure 5:**
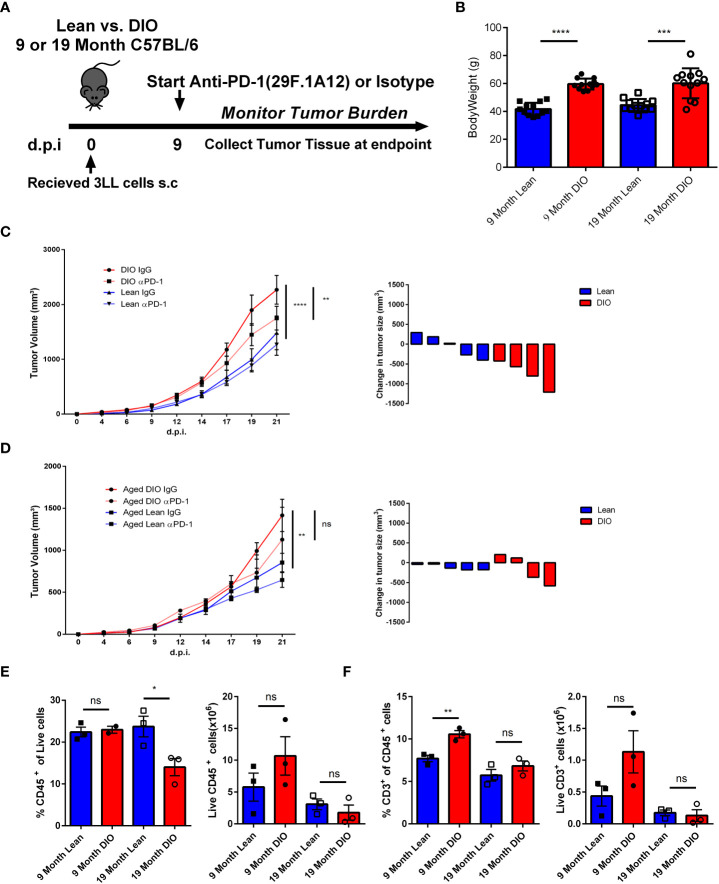
Aging Ameliorates the Increased Anti-Tumor Efficacy of ICI in DIO Mice. **(A)** Schema depicting experimental outline of nine- and nineteen-month-old lean and DIO mice challenged with subcutaneous 1x10^6^ 3LL tumor cells accompanied with anti-PD-1 checkpoint treatment or isotype control. **(B)** Baseline weights of nine and nineteen month lean and DIO mice (n= 12/group). **(C, D)** Tumor growth kinetics curves and waterfall plots depicting change in 3LL tumor volume following treatment in nine- and nineteen-month-old lean and DIO mice respectively (n = 4 – 6/group). **(E, F)** Representative flow cytometry results of CD45 and CD3 percent and total numbers respectively from processed 3LL tumor single cell suspensions (n =3/group). Graphs in **(C–F)** depict error bars based on s.e.m. Two-way ANOVA was used for analysis of **(C, D)**, unpaired student T test was used for assessments in **(B, E, F)**. *p < 0.05, **p < 0.01, ***p < 0.001, ****p < 0.0001, ns, not significant.

We have previously demonstrated that ICI using anti-PD-1 resulted in greater efficacy in six month DIO mice compared to lean diet mice using a variety of tumors and being confirmed clinically with anti-tumor efficacy responses (26). With this background and our observation that aged DIO mice displayed greater prevalence of classic T cell exhaustion markers in the periphery we wanted to explore the impact that aging may play in combination with obesity in modulating immunotherapy responses. It has been reported that there is decreased tumor cell line growth in murine models with advanced age and we determined if increased tumor progression in DIO mice occurred as has been observed by us and others (42–44). Twelve month lean and DIO mice were subcutaneously implanted with lung adenocarcinoma cells (3LL) and mice were assessed for tumor growth. From initial observations over the course of two weeks we noted that tumor growth was significantly greater in the DIO mice suggesting that even in age, obesity still promotes greater tumor progression. Once we established DIO tumors still grew faster with increased age, we next collected tumor tissue from twelve-month lean and DIO mice and performed RNA sequencing on the tumors. We investigated for key differences in immune exhaustive signatures, but surprisingly found minimal differences within the tumors in the lean and DIO mice at advanced age (Data not shown) even though increased peripheral T cell exhaustion was observed. To interrogate the efficacy of anti-PD-1 inhibition on anti-tumor responses in DIO mice, we compared male DIO mice at nine- and nineteen-months of age. We used an anti-PD-1 monotherapy regimen described previously in which younger DIO mice demonstrated significantly greater anti-tumor efficacy compared to lean control recipients (26) (**Figure 5A**). At baseline both nine- and nineteen-month-old mice demonstrated a significant differential in body weight between lean and DIO groups confirming that advanced age did not significantly decrease the bodyweight of DIO mice (**Figure 5B**). Additionally, assessment of peripheral T cells through flow cytometry revealed that even at the advanced age of nineteen months DIO mice still maintained elevated levels of PD-1 when compared to lean counterparts and more generally an increased exhaustive phenotype (**Supplemental Figure 4A**).

The tumor kinetic results illustrate that the nine- month DIO mice treated with anti-PD-1 demonstrated again increased efficacy with significant decreases in tumor volume compared to isotype-treated controls and lean mice experienced minimal impact of treatment in agreement with previous data and reports (**Figure 5C**). In marked contrast, the nineteen-month DIO mice receiving anti-PD-1 treatment, unlike their younger counterparts demonstrated insignificant anti-tumor responses (**Figure 5D**). This would be consistent with the increased exhaustion profile of the T cells we observed and the lack of differences in immune signatures observed in the tumors of the aged DIO and lean recipients. We then assessed the tumor infiltrate *via* flow cytometry and found increased immune cells within the tumor, notably T cells, in the younger DIO, but not in those of advanced age (**Figures 5E, F**). These observations suggest that although obesity can confer heightened efficacy with anti-PD-1 monotherapy in tumor models, this advantage can be mitigated by the additional immune modulatory impact of aging.

## Discussion

Our observations reiterate that the immune modulatory effects of obesity can exacerbate immune aging. We show that obesity both drives lymphopoiesis and engages thymic aging which results in enhanced memory conversion in DIO mice over lean controls. This T cell conversion escalates to the point that aged DIO mice have several fold greater increases in memory T cells than lean controls. Interestingly young DIO mice did not demonstrate significantly different exhaustive markers in peripheral blood at this earlier time point, but this changed markedly with increased age. In each case PD-1, Tim3 and Lag3 all were significantly increased in older DIO mice providing evidence that obesity and aging together is sufficient to induce an outwardly exhaustive phenotype in T cells. We show that regardless of age DIO mice demonstrate increased tumor progression. By immune phenotyping, we were able to delineate a state of terminal dysfunction induced by the combination of obesity and aging in which the increased efficacy of anti-PD-1 checkpoint blockade previously characterized in obesity is diminished and lost. Considering cancer is a disease that predominately afflicts those of increasing age the implications for this interplay and the different facets of inflammaging will need further study to determine which trends are clinically relevant and reflect patient outcomes.

We did not observe overt metabolic effects between aged lean and DIO mice despite being on HFD diet for months; we did not observe significant increases between six month and twelve-to-thirteen-month DIO resting glucose or hemoglobin A1c, but significant differences between lean and DIO mice at six months of age as previously reported (26) although it is likely the advanced age DIO mice are insulin-resistant which adds another variable regarding mechanisms contributing to immune dysfunction. More in-depth studies are needed to address the metabolic effects of this and other HFD diets over time on organ function and immune status. The differences in dieting systems including the use of HFD vs. western diets or the extent to which mice are maintained on these diets are well known and need to be considered when attempting to extrapolate preclinical modeling to the human condition as all likely contribute to effects. It will also be important to address sex-linked parameters on obesity-immune effects given reports that high BMI male patients responded with greater anti-tumor efficacy compared to high BMI females (44) as well as how age may influence outcome. Recent preclinical studies have indicated that sex-linked differences with obesity and immunotherapy can be observed (45) and it will be important to assess both aging and obesity over time as well as potential impact on the tumor type being modeled.

Defining the most appropriate preclinical modeling of obesity can be challenging. Obesity modeling can range from using mice strains genetically deficient in leptin or its receptor ob/ob and db/db mice respectively, which gain weight even when on normal diets, or by placing different strains of mice on specific high-fat diets (HFD) to induce obesity over time (46–48). While the former means can interrogate many question revolving around the lack of leptin or its receptor, they may also include aspects unrelated to body fat deposition. Use of a high fat diet (HFD) which often ranges from a composition of 45-60% fat-based calories is a common means of generating a DIO mouse and avoids complexities associated with a Western Diet which also includes high sugars. There are significant variables however, and include: the type of fat source, strain of the mouse used, the extent of time on the diet (which can range from weeks to months), the extent of weight gain determined to be considered “obese”, age when placed on the diet, as well as sex-linked effects. Metabolic parameters affected, such hyperglycemia or glucose intolerance can vary widely based on these parameters. Arguably, the DIO model is a better means of recapitulating the human obesity state versus the leptin deficient models which also can have developmental issues on immune and non-immune cell-types. The fact that obesity is a chronic condition that often goes on for years makes taking age into account of particular importance as our results indicate that increased immune effects are observed when obesity and aging are combined.

We have illustrated support for previous findings from Trottier et al. that the environment which obesity creates does promote thymopoiesis and the generation of a larger thymus, which is not simply a proportional increase based on the size of the mouse (21). These increases in cellular production are likely metabolically driven perhaps by growth factors including IGF1, which is known to stimulate lymphopoiesis (49, 50). However, this effect is time sensitive and after six-seven months in age thymic decline increases and thymic aging is accelerated. Yang et al. reported increased thymic involution being driven by obesity and on the surface it would appear to be at odds with the data presented by Trottier et al. (22). However, there were also important differences in the type of diet applied (45% HFD versus 60% HFD) as well as time on diet before assessment. Our data would suggest that obesity can indeed have dual effects on thymopoiesis which may be time-contingent but ultimately, a net negative effect can be associated with it due to both increased thymic involution as well as increased memory T cell conversion.

We have observed that obesity promotes increased T cell exhaustion and Treg activity and others have validated and connected the associated with greater tumorigenesis (35). And although some studies have had difficulty identifying exhaustion in DIO mouse models looking at the appropriate tissues is key (51); combined effects of obesity and the age of mice can dramatically influence the severity of exhaustion observed. We were able to observe a scaling effect in which obesity drastically increased in key exhaustive markers, which suggests with advanced age obesity may promote terminal exhaustion and dual expression of inhibitory markers including PD-1, Tim3 and Lag3 that can detrimentally affect the potential rescue of the cell (52, 53). When evaluating the implications of obesity’s impact on T cells in the context of cancer, we can first look at parallels with viral infection. Under infection obesity can lead to severe impairment of T cells particularly by rendering a memory response ineffective ultimately preventing adequate viral control (23, 24, 54, 55). In this study we observed that advanced age was able to impair efficacy of anti-PD-1 immune inhibition in aged obese mice that was otherwise effective in younger obese mice. Recent literature has confirmed similar findings with resistance to anti-PD-1 blockade in aged mice implicating suppression of TCR signaling, and defects in memory cell differentiation as potential causes (56).

Overall, with prevalence of obesity increasing in cancer patient populations, and with increased age being a factor in cancer occurrence, further and more comprehensive studies taking both these factors into account are needed (57). Better characterization of the dynamics of immune status in the context of aging along with obesity are required both to understand potential impairment of immunotherapies but also to better understand the biology of how inflammaging affects T cell priming, memory conversion, metabolism, and T cell effector function.

## Data availability statement

The data presented in the study are deposited in the NCBI BioProject repository, accession number PRJNA925221.

## Ethics statement

Animal studies were reviewed and approved by the Institutional Animal Care and Use Committees of either Roswell Park or the University of California Davis.

## Author contributions

LV and CC performed experiments, analyzed results, and co-wrote the article. ZW, LK and CD performed experiments and edited the article. SY, RS, KS performed experiments and data analysis. JB designed experiments, performed data analysis, and edited the article. EA, AM, and RC edited the article. WM directed the project, designed experiments, interpreted results, and co-wrote the article. All authors contributed to the article and approved the submitted version.

## Supplementary material

Supplemental Figure 1 Lean and DIO thymic Cellularity and CD4/CD8 Sub populations. (A) Thymic cellularity of six-seven, twelve-thirteen-month, or sixteen-nineteen-month male C57BL/6 mice plotted with corresponding total body weight with linear interpolation (B) Lean and DIO thymic sub population percentages assessed via flow cytometry and gated on CD4 single positive, CD8 single positive and CD4/CD8 double negative thymocytes (n = 4-5). (C) Total Numbers of lean and DIO thymic sub populations obtained though assessment of flowcytometry interrogation in combination with overall thymic cellularity (n = 4-5). (E) Depiction of percent Annexin V positive lean and DIO thymic sub populations (n = 4-5). (F) total numbers of Annexin V positive cells of lean and DIO thymic sub populations CD4 single positive, CD8 single positive and CD4/CD8 double negative (n = 4-5). Graphs depict error bars based on standard deviation. Data are representative of at least two independent experiments. Simple linear regression and a runs test was used in (C). Unpaired student T test was used for assessments in (B-F). *p < 0.05, **p < 0.01, ***p < 0.001, ****p < 0.0001, ns, not significant.

Supplemental Figure 2 Lean and DIO memory compartments with elevated age. (A) Flow cytometry staining of CD44 and CD62L +/- frequencies from the peripheral blood of lean control or Diet induce obese (DIO) five and eleven-month-old C57BL/6 mice; percentages of effector memory (CD44+ CD62L-), central memory (CD44+ CD62L+), Naïve (CD44- CD62L+) and double negative (CD44- Cd62L-) T cell memory subsets are displayed (n = 3-4/group). (B) Fold change in in respective T cell memory subsets across increases in age in lean and DIO mice. Graphs depict error bars based on standard deviation. Data are representative of at least two independent experiments.

Supplemental Figure 3 Memory T cells exhibit elevated exhaustive markers. (A) Flow cytometry frequencies of PD-1+, TIM3+ and Lag3 + on memory (CD44+) CD3 T cells from the peripheral blood of lean control or DIO five and eleven-month-old C57BL/6 mice (n = 3-4/group). B) Flow cytometry gating strategy used for T cell analysis including evaluation of Treg and memory subsets. Graphs depict error bars based on standard deviation. Data are representative of at least two independent experiments. Unpaired student T test was used for assessments in (A). *p < 0.05, **p < 0.01, ***p < 0.001, ****p < 0.0001, ns, not significant.

Supplemental Figure 4 Effects of HFD on weight gain and metabolism over time. (A) Flowcytometry assessment of exhaustion markers PD-1, Lag3 and Tim3 in the splenic T cells of nineteen-month-old C57BL/6 lean and DIO mice (n = 4-8/group). Graphs depict error bars based on standard deviation. Data are representative of at least two independent experiments. Unpaired student T test was used for assessments in (A). *p < 0.05, **p < 0.01, ***p < 0.001, ****p < 0.0001, ns, not significant.
