# Supplementary material for: Aging augments obesity-induced thymic involution and peripheral T cell exhaustion altering the “obesity paradox”
Source: Front Immunol. 2023 Jan 26;13:1012016. doi: 10.3389/fimmu.2022.1012016 (PMC9910174; doi:10.3389/fimmu.2022.1012016)
Supplement: Supplemental Figure 1 — Lean and DIO thymic Cellularity and CD4/CD8 Sub populations. (A) Thymic cellularity of six-seven, twelve-thirteen-month, or sixteen-nineteen-month male C57BL/6 mice plotted with corresponding total body weight with linear interpolation (B) Lean and DIO thymic sub population percentages assessed via flow cytometry and gated on CD4 single positive, CD8 single positive and CD4/CD8 double negative thymocytes (n = 4-5). (C) Total Numbers of lean and DIO thymic sub populations obtained though assessment of flowcytometry interrogation in combination with overall thymic cellularity (n = 4-5). (E) Depiction of percent Annexin V positive lean and DIO thymic sub populations (n = 4-5). (F) total numbers of Annexin V positive cells of lean and DIO thymic sub populations CD4 single positive, CD8 single positive and CD4/CD8 double negative (n = 4-5). Graphs depict error bars based on standard deviation. Data are representative of at least two independent experiments. Simple linear regression and a runs test was used in (C). Unpaired student T test was used for assessments in (B-F). *p < 0.05, **p < 0.01, ***p < 0.001, ****p < 0.0001, ns, not significant. [file DataSheet_1.pdf]

Title: Aging Augments Obesity-Induced Thymic Involution and Peripheral T cell Exhaustion Altering the "Obesity Paradox"

Supplemental Figures and Information

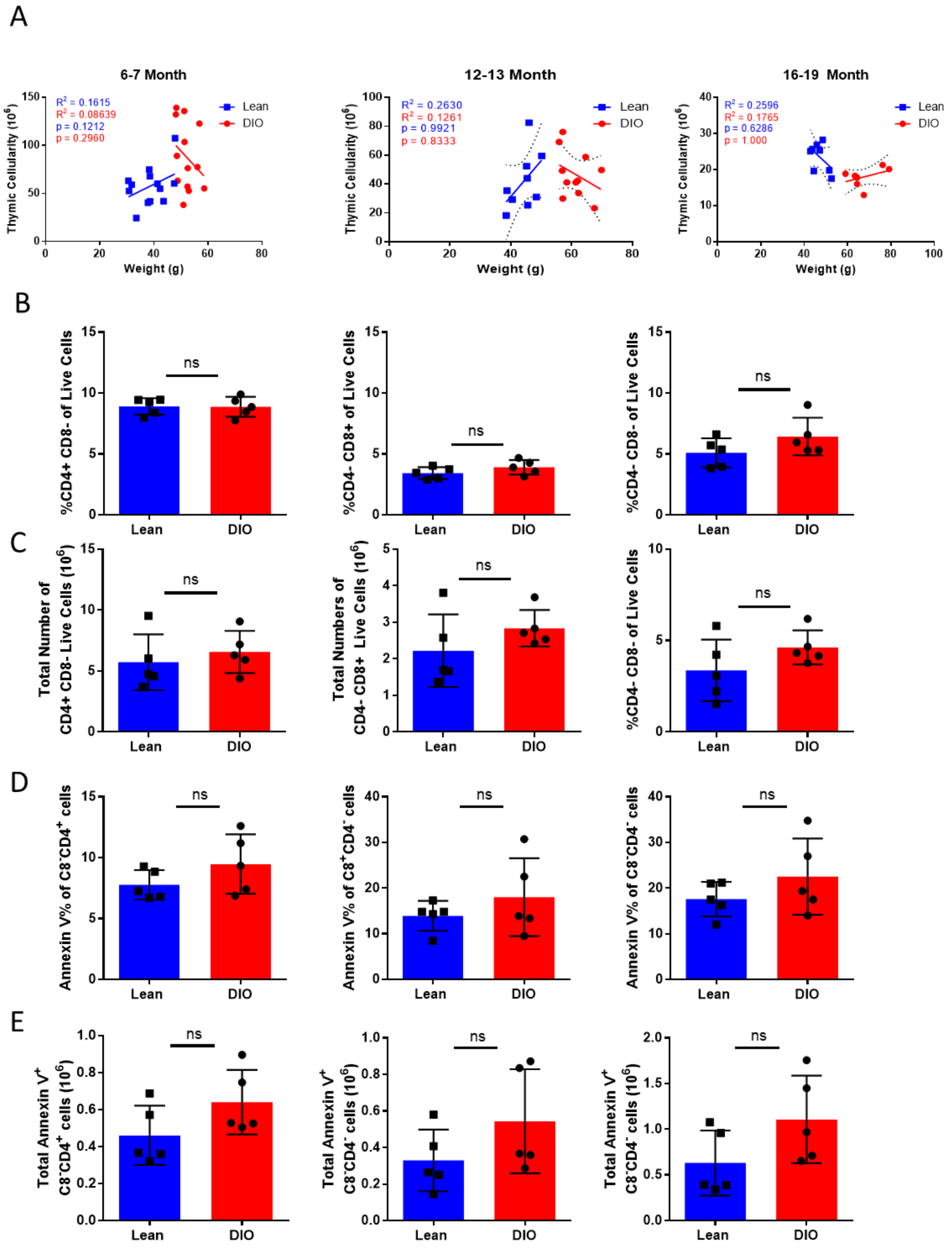

Supplemental Figure 1: Lean and DIO thymic Cellularity and CD4/CD8 Sub populations. (A) Thymic cellularity of six-seven, twelve-thirteen-month, or sixteen-nineteen-month male C57BL/6 mice plotted with corresponding total body weight with linear interpolation (B) Lean and DIO thymic sub population percentages assessed via flow cytometry and gated on CD4 single positive, CD8 single positive and CD4/CD8 double negative thymocytes (n = 4-5). (C) Total Numbers of lean and DIO thymic sub populations obtained though assessment of flowcytometry interrogation in combination with overall thymic cellularity (n = 4-5). (E) Depiction of percent Annexin V positive lean and DIO thymic sub populations (n = 4-5). (F) total numbers of Annexin V positive cells of lean and DIO thymic sub populations CD4 single positive, CD8 single positive and CD4/CD8 double negative (n = 4-5). Graphs depict error bars based on standard deviation. Data are representative of at least two independent experiments. Simple linear regression and a runs test was used in (C). Unpaired student T test was used for assessments in (B-F). $^{*}p < 0.05$, $^{**}p < 0.01$, $^{***}p < 0.001$, $^{****}p < 0.0001$, ns, not significant.

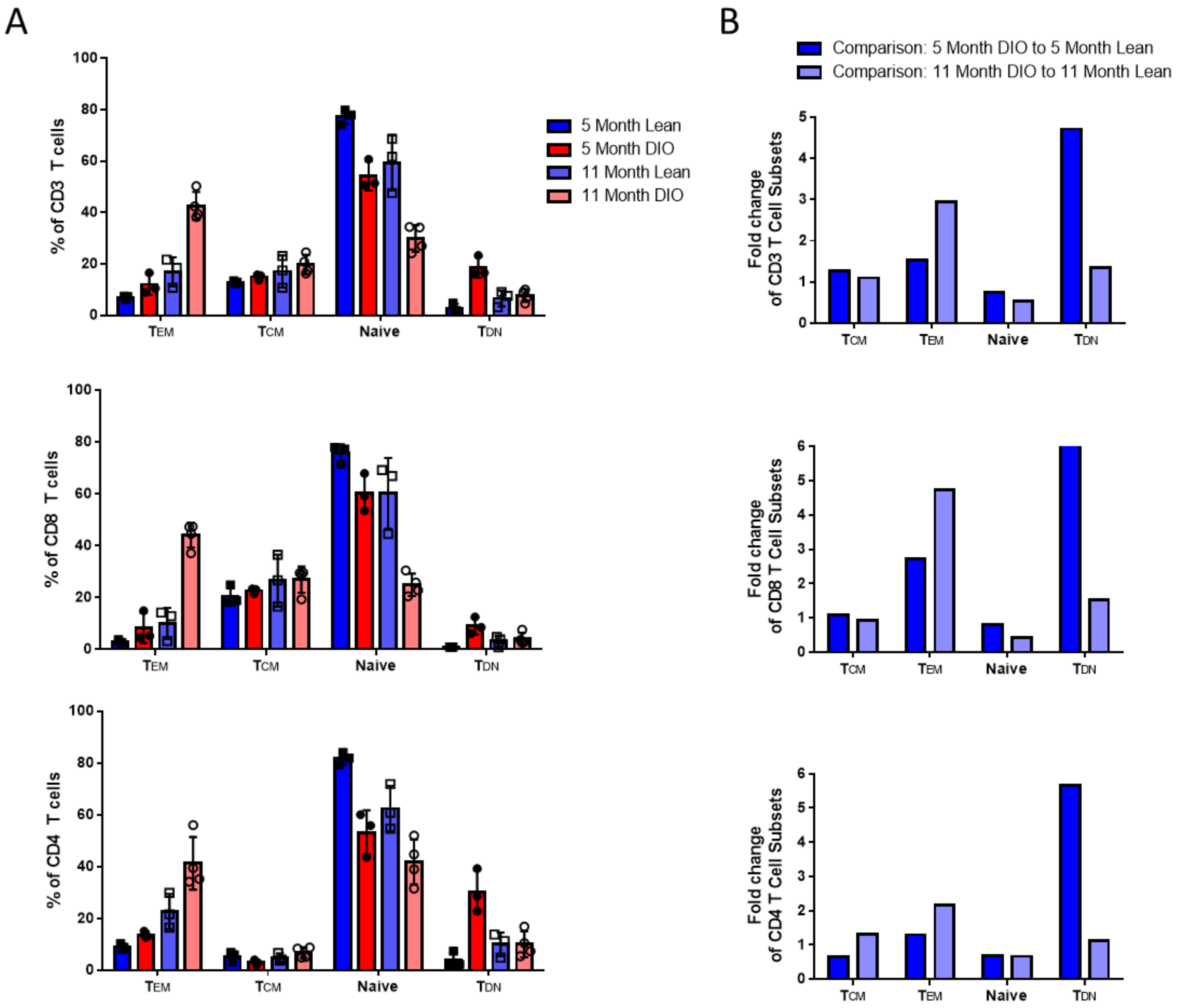

Supplemental Figure 2: Lean and DIO memory compartments with elevated age. (A) Flow cytometry staining of CD44 and CD62L +/- frequencies from the peripheral blood of lean control or Diet induce obese (DIO) five and eleven-month-old C57BL/6 mice; percentages of effector memory (CD44+ CD62L-), central memory (CD44+ CD62L+), Naïve (CD44- CD62L+) and double negative (CD44- Cd62L-) T cell memory subsets are displayed (n = 3-4/group). (B) Fold change in in respective T cell memory subsets across increases in age in lean and DIO mice. Graphs depict error bars based on standard deviation. Data are representative of at least two independent experiments.

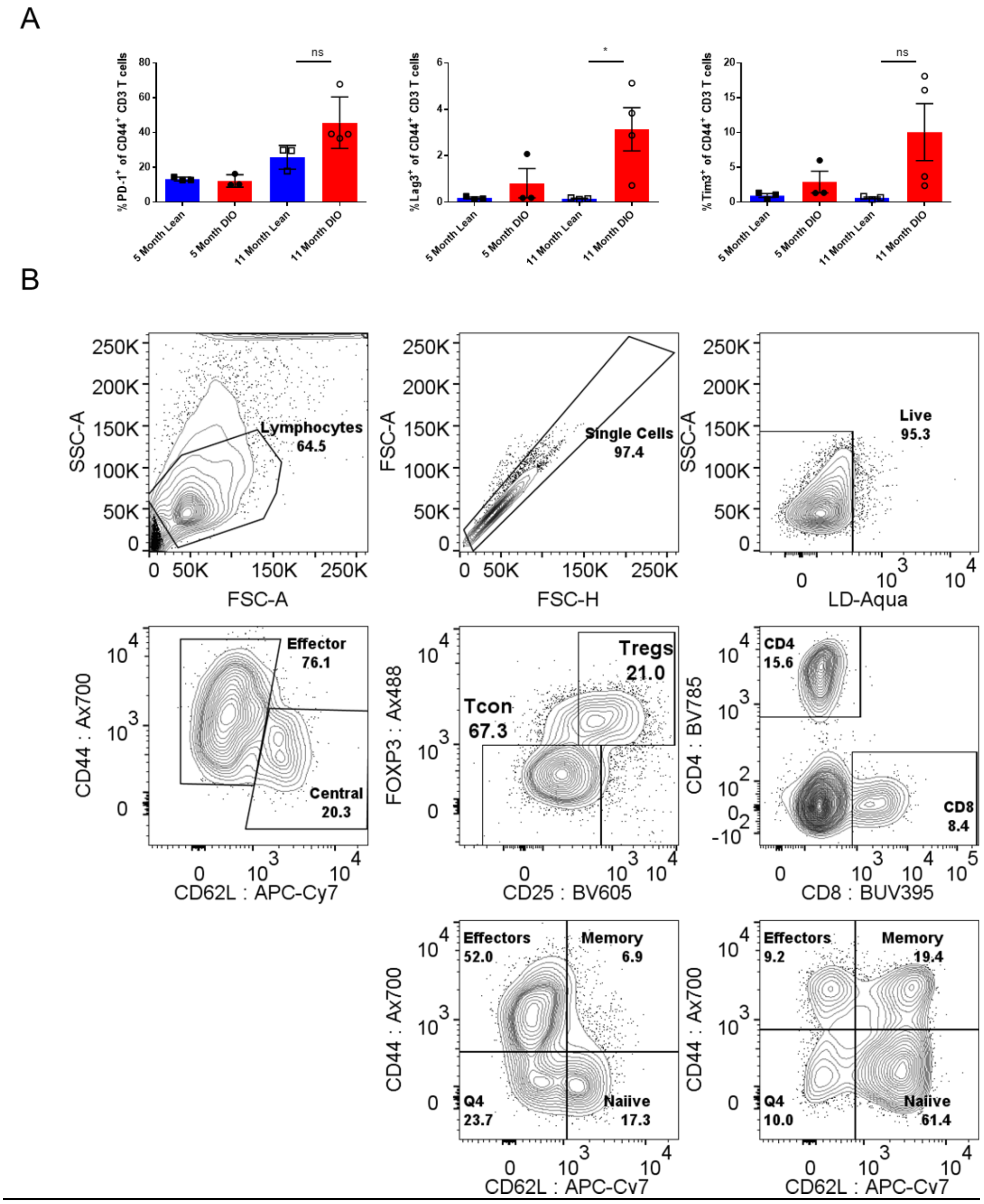

Supplemental Figure 3: Memory T cells exhibit elevated exhaustive markers. (A) Flow cytometry frequencies of PD-1^+, $TIM3^+$ and Lag3 $^+$ on memory (CD44+) CD3 T cells from the peripheral blood of lean control or DIO five and eleven-month-old C57BL/6 mice (n = 3-4/group). B) Flow cytometry gating strategy used for T cell analysis including evaluation of Treg and memory subsets. Graphs depict error bars based on standard deviation. Data are representative of at least two independent experiments. Unpaired student T test was used for assessments in (A). *p < 0.05, **p < 0.01, ***p < 0.001, ****p < 0.0001, ns, not significant.

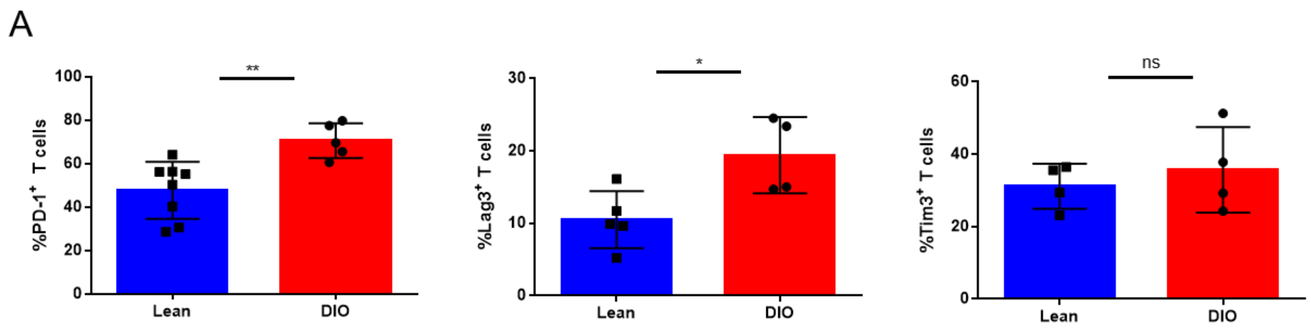

Supplemental Figure 4: Effects of HFD on weight gain and metabolism over time. (A) Flowcytometry assessment of exhaustion markers PD-1, Lag3 and Tim3 in the splenic T cells of nineteen-month-old C57BL/6 lean and DIO mice (n = 4-8/group). Graphs depict error bars based on standard deviation. Data are representative of at least two independent experiments. Unpaired student T test was used for assessments in (A). *p < 0.05, **p < 0.01, ***p < 0.001, ****p < 0.0001, ns, not significant.

Supplemental Table 1: Control Diet Bodyweight and Thymic Cellularity

Strain	Sex	Age (Months)	Diet	Bodyweight (grams)	Thymic Cellularity (10^6 Cells)
C57BL/6	Male	6	10% Fat	43.6	42.1225
C57BL/6	Male	6	10% Fat	37.8	40.4275
C57BL/6	Male	6	10% Fat	42.3	55.1
C57BL/6	Male	6	10% Fat	33.5	24.58
C57BL/6	Male	6	10% Fat	38.3	74.93
C57BL/6	Male	6	10% Fat	31.8	59.19
C57BL/6	Male	7	10% Fat	30.6	63.13
C57BL/6	Male	7	10% Fat	30.8	52.54
C57BL/6	Male	7	10% Fat	41.3	60.195
C57BL/6	Male	7	10% Fat	47.5	60.44
C57BL/6	Male	7	10% Fat	38.6	41.92
C57BL/6	Male	7	10% Fat	38.5	67.975
C57BL/6	Male	7	10% Fat	47.8	107.5
C57BL/6	Male	12	10% Fat	48.4	31.15
C57BL/6	Male	12	10% Fat	45.6	25.47
C57BL/6	Male	12	10% Fat	38.8	35.42
C57BL/6	Male	12	10% Fat	45.4	44.01
C57BL/6	Male	13	10% Fat	38.5	18.2725
C57BL/6	Male	13	10% Fat	40.5	29.3425
C57BL/6	Male	13	10% Fat	45.2	52.35
C57BL/6	Male	13	10% Fat	46	82.53
C57BL/6	Male	13	10% Fat	50.2	59.57
C57BL/6	Male	16	10% Fat	42.8	25.115
C57BL/6	Male	16	10% Fat	45.8	26.875
C57BL/6	Male	16	10% Fat	43.8	25.705
C57BL/6	Male	16	10% Fat	47.2	25.365
C57BL/6	Male	16	10% Fat	51.6	19.83
C57BL/6	Male	19	10% Fat	52.6	17.55
C57BL/6	Male	19	10% Fat	48.5	28.26
C57BL/6	Male	19	10% Fat	44.4	19.655

Supplemental Table 2: HFD Bodyweight and Thymic Cellularity

Strain	Sex	Age (Months)	Diet	Bodyweight (grams)	Thymic Cellularity (10^6 Cells)
C57BL/6	Male	6	60% Fat	52.4	57.225
C57BL/6	Male	6	60% Fat	50.9	38.235
C57BL/6	Male	6	60% Fat	52.9	52.75
C57BL/6	Male	6	60% Fat	48.1	132.26
C57BL/6	Male	6	60% Fat	48.5	89.17
C57BL/6	Male	6	60% Fat	51.3	103.73
C57BL/6	Male	7	60% Fat	51.2	135.75
C57BL/6	Male	7	60% Fat	48.3	139.2
C57BL/6	Male	7	60% Fat	56.13	77.6
C57BL/6	Male	7	60% Fat	56.81	122.8
C57BL/6	Male	7	60% Fat	48.8	63.305
C57BL/6	Male	7	60% Fat	58.6	55.33
C57BL/6	Male	7	60% Fat	52.5	76.39
C57BL/6	Male	12	60% Fat	57.1	76.11
C57BL/6	Male	12	60% Fat	56.9	49.33
C57BL/6	Male	12	60% Fat	57.1	30.045
C57BL/6	Male	12	60% Fat	62	42.395
C57BL/6	Male	13	60% Fat	67.5	23.38
C57BL/6	Male	13	60% Fat	64.6	58.775
C57BL/6	Male	13	60% Fat	58.4	41.19
C57BL/6	Male	13	60% Fat	69.8	49.78
C57BL/6	Male	13	60% Fat	61.4	41.21
C57BL/6	Male	13	60% Fat	62.3	33.81
C57BL/6	Male	13	60% Fat	55.9	69.24
C57BL/6	Male	16	60% Fat	59	18.87
C57BL/6	Male	16	60% Fat	76.2	21.33
C57BL/6	Male	16	60% Fat	64.4	16.065
C57BL/6	Male	16	60% Fat	63.4	18.305
C57BL/6	Male	16	60% Fat	64.4	17.82
C57BL/6	Male	19	60% Fat	79.2	20.16
C57BL/6	Male	19	60% Fat	63.8	18.06
C57BL/6	Male	19	60% Fat	67.5	12.98
